# Linking theory and practice to advance sustainable healthcare: the development of maturity model version 1.0

**DOI:** 10.1186/s12913-024-11749-8

**Published:** 2024-11-05

**Authors:** Marieke Sijm-Eeken, Hans C. Ossebaard, Aleksandra Čaluković, Bram Temme, Linda W. Peute, Monique W. Jaspers

**Affiliations:** 1 Department of Medical Informatics, Center for Sustainable Healthcare, Amsterdam Public Health Research Institute, Amsterdam UMC Location University of Amsterdam, Meibergdreef 9, Amsterdam, The Netherlands; 2 National Health Care Institute, Diemen, The Netherlands; 3 Athena Institute, Vrije Universiteit Amsterdam, Amsterdam, The Netherlands; 4 Department of Medical Informatics, Amsterdam UMC Location University of Amsterdam, Meibergdreef 9, Amsterdam, The Netherlands; 5 Department of Medical Informatics, Center for Human Factors Engineering of Health Information Technology, Amsterdam Public Health Research Institute, Amsterdam UMC Location University of Amsterdam, Meibergdreef 9, Amsterdam, The Netherlands

**Keywords:** Maturity model, Sustainability, Climate change, Healthcare, Self-assessment, Quality improvement

## Abstract

**Background:**

Climate change and increased awareness of planetary health have made reducing ecological footprints a priority for healthcare organizations. However, improving healthcare’s environmental impact remains difficult. Numerous researchers argue these difficulties are caused by healthcare’s environmental impact being multidimensional, influenced throughout the healthcare chain, and often has downstream consequences that are hard to identify or to measure. Even though existing research describes many successful approaches to reduce healthcare’s environmental impact, a robust multidimensional framework to assess this impact is lacking. This research aims at developing a maturity model for sustainable healthcare that could be used for self-assessment by healthcare professionals to identify improvement actions and for sharing best practices in environmental sustainability.

**Methods:**

A design-oriented approach for maturity model development was combined with an expert panel and six case studies to develop, refine and expand the maturity model for environmentally sustainable healthcare.

**Results:**

A maturity model was developed containing four domains: ‘Governance’, ‘Organization Structures’, ‘Processes’, and ‘Outcomes and Control’. Applying the model in real-world environments demonstrated the model’s understandability, ease of use, usefulness, practicality and ability to identify improvement actions for environmental sustainability in healthcare organizations.

**Conclusions:**

This study found that healthcare practitioners could apply the maturity model developed and tested in this study in several hours without training to help them gain valuable insights into the environment footprint of the healthcare setting they worked in.

Systematically implementing the model developed in this study could help address the urgent need to mitigate the substantial environmental impact of healthcare. These implementations can help evaluate and improve the maturity model.

**Supplementary Information:**

The online version contains supplementary material available at 10.1186/s12913-024-11749-8.

## Background

Climate change and planetary health awareness have made lowering their ecological impact a new and competitive priority in modern healthcare organizations [1, 2]. This is only a recent development and healthcare’s environmental and climate impact are still substantial. According to the 2023 Lancet Countdown Report, healthcare emissions comprise 4-6% of global greenhouse gas emissions, and healthcare-associated air pollution by particulate matter and ozone is estimated to cause around 4 million Disability-Adjusted Life Years yearly [3].

Improving the necessary environmental sustainability of healthcare is a difficult task. The environmental impact of healthcare is complex and encompasses the entire healthcare chain at multiple levels including the national health system and global supply chain levels [4, 5]. Moreover, healthcare’s impact often leads to distal impacts that may not be immediately apparent within the organization that causes the impact [6–8]. Initiatives aimed at implementing sustainable solutions face a range of technological and organizational challenges [9].

To date, research has considered several useful methods, tools and practices to address healthcare’s environmental footprint. Examples include Life Cycle Assessment studies [10], circular economy practices [11], standardized sustainability performance metrics [5], Green Six Sigma [12] and the use of information technology [13]. Since existing methods appear to be time consuming or too complex to be used by healthcare professionals without extensive training, their usage and effect are suboptimal. Therefore, in this study we seek to develop a more efficient and effective approach to support healthcare professionals with assessing the current status of sustainability and identifying improvement options to reduce environmental impact of their organizations.

A maturity model could offer these functionalities. Maturity models are valuable tools that specify simplified stages or levels of maturity, for evaluating the present level of development in a certain area of an organization and supporting identification of areas of strength and areas that require improvement [14, 15].

The purpose of this study is to develop a maturity model for sustainable healthcare. The aim of the model is twofold. Firstly, to support healthcare organizations and their various components, such as divisions or departments, in evaluating their progress towards reducing their environmental footprint. Therefore, the model should facilitate gaining insights on the current state of basic concepts that contribute to establishing an environmentally sustainable healthcare organization. Additionally, it should assist in the identification of possible steps for achieving a higher level of sustainability in terms of healthcare organizations environmental impact. Sustainability in terms of improving climate resilience, is not included in this first version of the model. The second aim of the model is to foster mutual learning and improvement by facilitating benchmarking amongst healthcare organizations or their departments.

## Methods

**Fig. 1:**
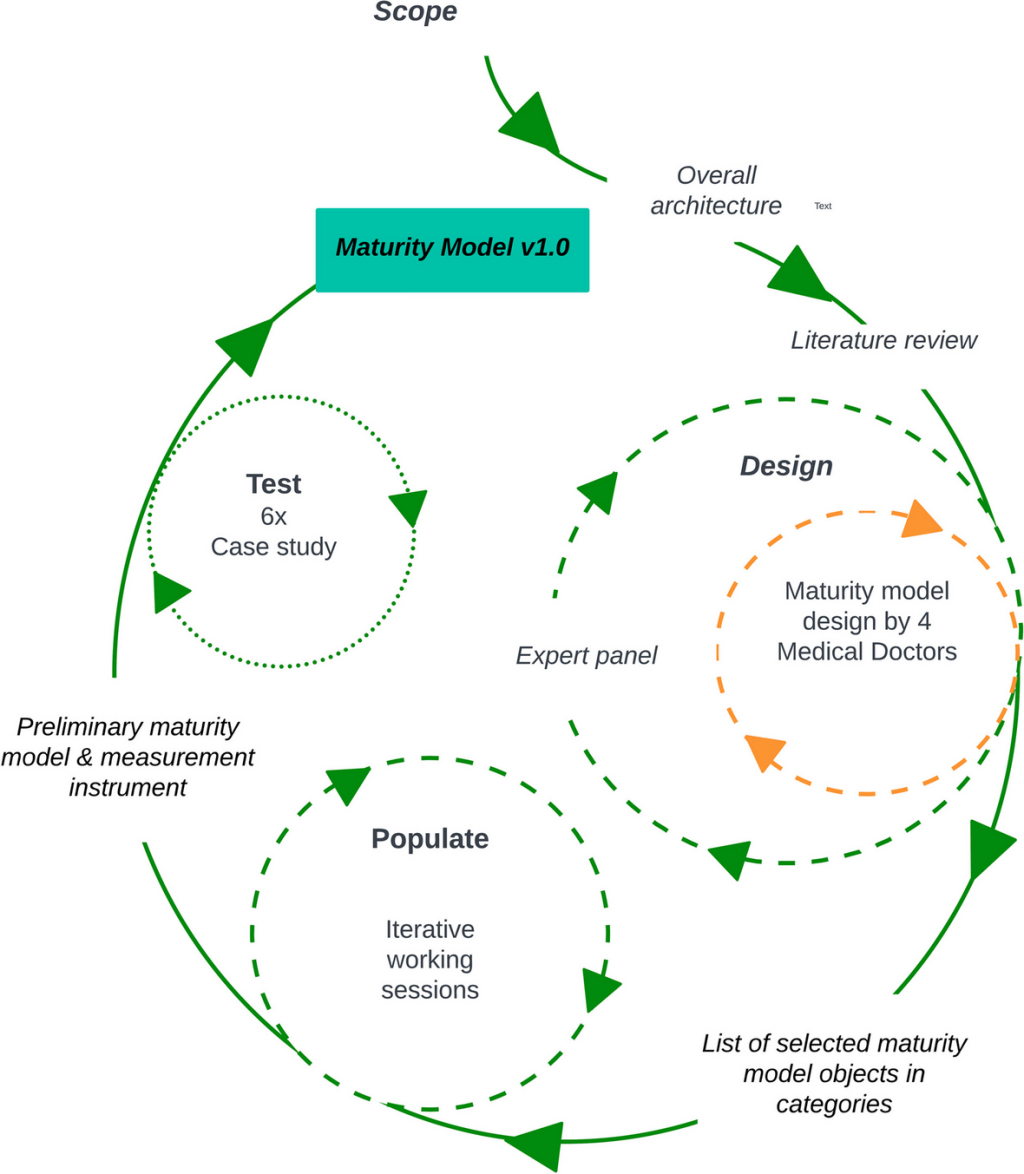
Overall research design

There is currently no prevailing preferred approach for the development of maturity models. Typical methodologies involve utilizing established maturity models as a foundational framework and applying design-oriented approaches [15]. Testing the model is an essential step when developing a maturity model, to ascertain the practicality and advantages in real-life situations [15]. In the present study we apply the design-oriented methodology outlined by De Bruin et al. [16] to construct a maturity model for sustainable healthcare. The approach includes a test phase and is one of the few structured approaches for developing new maturity models available in scientific literature. The methodology consists of the following phases: scope, design, populate, test, deploy and maintain. The latter two phases can be accomplished when the model has been made available to a wider audience which is an objective of the current paper. Consequently, these phases are not included in this publication. The overall research design of the present study is shown in Fig. 1. The following sections provide further details on each phase.

### Scope phase

The scope of the model is defined by the demand from healthcare professionals involved in reducing environmental impact of a healthcare organization. The focus was set to the specific domain of sustainability in terms of environmental impact of healthcare organizations, in the Dutch context. Since the research team that developed the model consisted of both scientists and healthcare professionals involved in sustainable healthcare in the Netherlands, the model to be developed was viewed as an evidence-based framework with a strong practical focus.

### Design phase

In the design phase the model’s structure and content are developed. The design decisions agreed upon by the research team are presented in Appendix A, table A1. The overall architecture of the model was defined by the research team, following the design process as proposed by De Bruin et al. [16] with five progressive stages where higher stages are built upon the requirements of lower stages. Level 1 represents a low level of maturity, while level 5 signifies the highest level of maturity.

The content of the model was defined by using a mixed-method approach consisting of a literature review, a parallel design project by medical doctors, an expert panel review and a final synthesis by the research team. The expert panel consisted of two academia, three healthcare practitioners, an environmental expert and a policy maker all involved and experienced in environmental sustainability improvement in healthcare.

The literature review aimed to identify relevant concepts from the domain of sustainable healthcare and the relevant enabling and impeding factors to be included in the model. Because this domain was relatively new at the time of constructing the maturity model, a systematic literature review would not be an efficient method for identifying these concepts. Instead, a RAPID review was conducted based on the Cochrane Rapid reviews methods group [17]. A summary of the RAPID review protocol can be found in Appendix B. Records identified by running the search query were imported into Rayyan.ai web application to screen titles and abstracts by two researchers. In an independent selection process, papers were identified for inclusion and subsequently analyzed to extract key concepts and measurement criteria. Differences were discussed and resolved between the researchers.

To supplement the limited theoretical basis of the emerging field of sustainable healthcare maturity, a parallel subproject was conducted with four medical doctors with diverse professional backgrounds to design a maturity model for sustainable healthcare. They participated in an 8-week masterclass on sustainability in healthcare as part of a two-year MBA program at the University of Amsterdam. In parallel with the literature review, they designed their own version of a maturity model for sustainable healthcare by using their perspectives and experiences as medical doctors as well as any other materials they wanted to use such as educational materials from the masterclass or scientific literature.

After completing the literature review and the design by medical doctors, concepts with measurement criteria resulting from both research steps were combined and consolidated into a structured overview of unique concepts organized in groups (concept categories).

Subsequently, an expert panel reviewed the structure to determine the optimal set of concepts and concept categories to include in the maturity model. The expert panel consisted of two scientists, three healthcare practitioners, an environmental expert and a healthcare policy advisor, all experienced in environmental sustainability improvement in healthcare. In accordance with the design principle on minimizing the number of objects from De Bruin et al. [16], the expert panel was asked to select the most important concept categories and concepts to minimize complexity and ensure independence of model components (eg. to prevent overlap between definitions of concepts). The selection resulted in an overview of potential concepts with measurement criteria organized in categories to form the domains of the maturity model.

### Populate phase

A preliminary maturity model was constructed, utilizing the conceptual categories selected by the expert panel. These concept categories formed the domains in which the concepts are categorized. Furthermore, a measurement instrument was developed to support the documentation of maturity levels for each concept (or: maturity model object) and to automatically provide maturity level averages per domain (concept category). This instrument was designated as the “Sustainable Healthcare Maturity Checklist” and is alternatively referred to as “the checklist.” It was generated through an iterative process conducted by the research team over several working sessions.

### Test phase

The efficacy of the model and its checklist were evaluated by implementing them in case studies from different healthcare settings, while assessing their ease of understanding, ease of use, usefulness, and practicality with the individuals involved in implementing the model.

Six case studies were chosen based on availability and location (within or near Amsterdam, the Netherlands) to allow for actual attendance by junior researchers during the assessment in June 2023. After receiving a briefing on the model’s aim and structure, different researchers with no prior knowledge or experience with the maturity model applied the model to the case study assigned to them. Five case studies focused on a department (Gynecology, Surgery, Psychiatry, Pediatric surgery, and Neonatal care) within the Amsterdam University Medical Center and one in a geriatric care institute in the city of Purmerend. The researchers independently reviewed the maturity model checklist and collected data over a two- to five-hour site visit to determine each checklist item’s maturity level. Researchers rated each checklist item in conjunction with department staff, particularly nurses and doctor’s assistants. The ratings were determined by considering input from department staff, observing process workflows, analyzing reports and (electronic) documentation such as management dashboards and product ordering history. If there were any remaining inquiries, supervisors or facilitating staff members were contacted.

Maturity checklist scores were reviewed and agreed upon by the researchers with one or more staff members involved in daily practice at the respective organization/department. Strengths (higher maturity level scores) and improvement areas (lower maturity level scores) were identified, and improvement actions resulting from the model were discussed accordingly. To define these improvement actions, particular attention was given to the lower scores. A cause & effect analysis was used to ensure that actions were targeted and effective. This process involved two main steps: first, identifying the root causes of the low scores, and then brainstorming potential solutions. Researchers played a facilitative role by asking probing questions, such as “Why do you think this score was low?” or “What challenges might be contributing to this issue?” This helped the staff to dig deeper into the underlying problems. After identifying the root causes, the team collectively brainstormed various solutions, considering the feasibility and practicality of each option. The most viable solutions were selected for implementation, ensuring that the improvement actions were both realistic and directly addressed the root causes identified. This collaborative and systematic approach ensured that the improvement actions were well-grounded and had a higher likelihood of leading to meaningful changes.

A survey based on the form for evaluating maturity models from Salah et al. [18] was then used to assess the model’s understandability, ease of use, usefulness, practicality and ability to identify improvement actions for environmental sustainability in healthcare organizations. The maturity model evaluation survey is presented in Appendix C. The survey was completed by the researchers assigned to each case study after applying the maturity model. Survey findings were consolidated and aggregated to determine scores given to each evaluation criterion. Statistical analysis was applied to test on correlation between criterion and differences between departments in R (version 4.3.1.), using a parametric ANOVA test for categories with normal distribution and similar variances and a non-parametric Kruskal–Wallis test for the remaining categories. The answers to open questions were consolidated and analyzed via deductive coding using ATLAS.ti (version 23.3.4.) by two researchers independently and by using the themes of the questionnaire as the coding structure. Differences between coding were resolved by discussion between the researchers. Findings from the surveys were accordingly evaluated by the research team to identify improvements to be made to the maturity model and instrument.

## Results

The maturity stages defined for the sustainable healthcare model’s overall architecture with their labels and description following the top-down design based on De Bruin et al. [16] are provided in Table 1.

**Table 1:** Stages of maturity with description defined by research team

**Maturity level**		**Description**
1	Initiated	Little to no sustainability focus with unclear focus and lack of tools and support
2	Developing	Develop sustainable strategy and define ad hoc initiatives
3	Defined	Practices are documented and standardized
4	Measured	Sustainable processes formalized and measured, top-down and bottom-up focus combined
5	Optimized	Sustainable processes are optimized with structured monitoring of outcomes and performance with control mechanisms

**Fig. 2:**
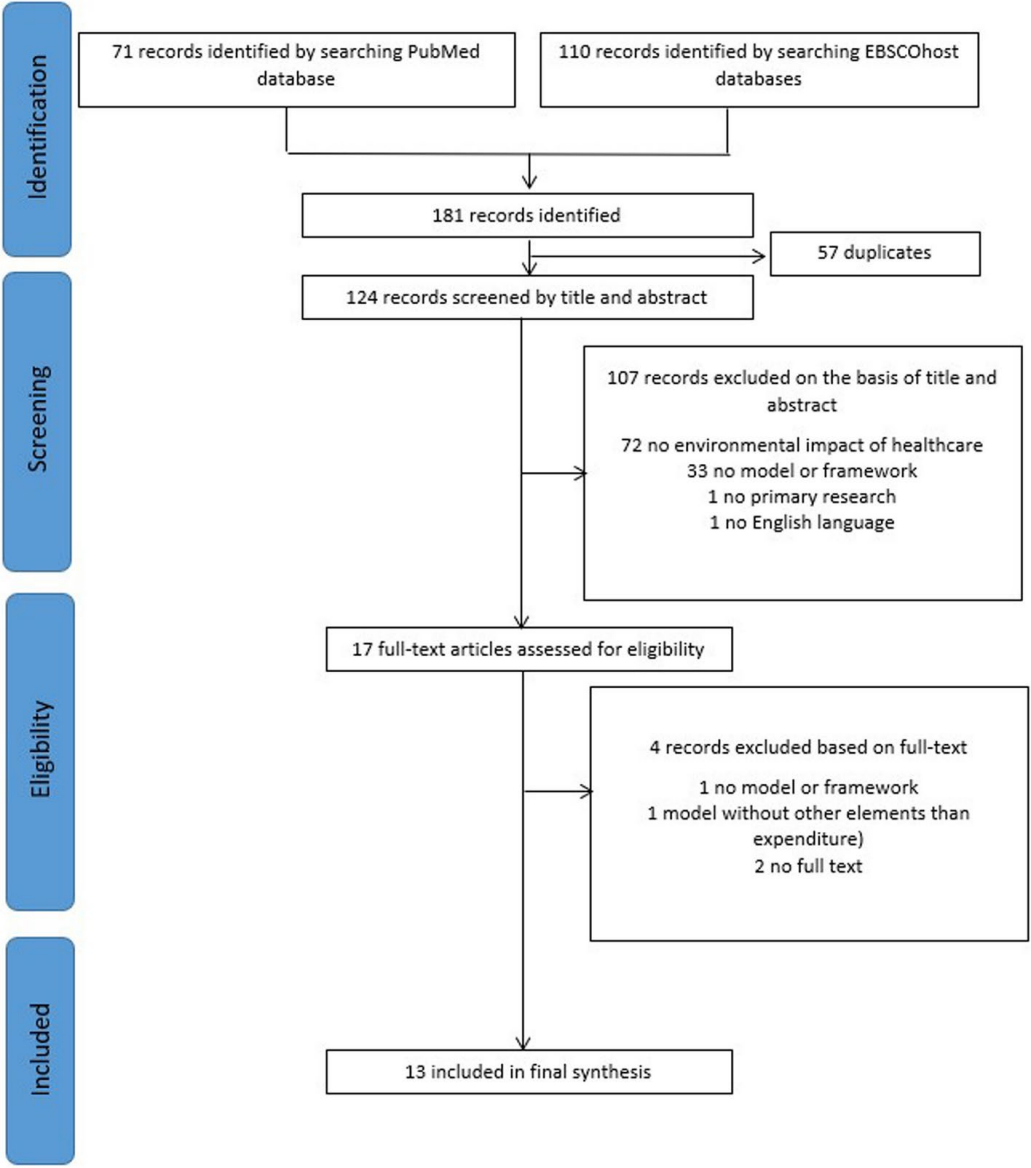
PRISMA diagram summarizing literature review process

The screening and selection process followed for the literature review is summarized in Fig. 2.

The concepts extracted from the thirteen articles resulting from the literature search are presented in Table 2 together with the concepts identified in the parallel sub-project by medical doctors. All concepts are related to sustainability in healthcare. The decision of the expert panel for including the concepts in the maturity model can also be found in this table.

**Table 2:** Overview of identified (sub)concepts related to sustainable healthcare with source and decision on inclusion

**Concept category (Domain)**	**Concept**	**Subconcept**	**Include in Maturity Model?**
External	Laws and regulations	Laws^a^ [19, 20] Regulations [19, 20]	No
	Partnerships	Collaboration with other institutions to influence government policy [19]	No
	Community building	Share knowledge and experience with other organisations^a^	No
Governance	Vision on sustainability	Focus on long term impacts in decision making [19] Sense of obligation to be a leader within the community [19] Environmental sustainability integrated in identity and mission [19]	Yes
	Time and budget	Investment in roles dedicated to sustainability^a^ [19]	Yes
Structure	Person(s) / Staff	Visitor travel [21–26] Patient travel [21–26, 30] Staff commute [21–26] Awareness on sustainability^a^ [19, 23] Teams or departments dedicated to sustainability [19] Stakeholder engagement^a^ [23]	Yes
	Facilities design/building	Building construction [22]	No
		Infrastructure^a^ [20]	Yes
	Tools and technology	Tools for waste management to support effective separation of waste^a^ Use of technology and innovation to realize more environmentally sustainable healthcare practices^a^	Yes
		Medical Equipment [20–22, 24] Non-medical equipment^a^ [20–22, 24] ICT [22, 26] Telemedicine [23, 26] MRI [27]	Yes^b^
	Culture	Beliefs [19] Feelings of importance [19] Feelings of responsibility [19] Commitment for driving change^a^ [19] Staff feels comfortable initiating change^a^ [19] Shared desire to inspire other employees and institutions [19] Ambition to receive awards and recognition^a^ [19] Executive Support of staff ideas^a^ [19]	Yes
	Knowledge	Level of knowledge on sustainable healthcare^a^ Availability of training on sustainable healthcare^a^	Yes
	Readiness for change	Executive or organizational support^a^ [19] Processes and procedures are conductive to change and innovation [19]	Yes
Process	Improvement projects	Sustainability improvement projects [23]	Yes
	Waste management	Waste products and recycling [20–23, 27, 28] Diagnostic imaging waste [27]	Yes
	Pollution air/water/land	Anaesthetic gases and medical dose inhalers [21, 22, 28, 29]	Yes
	Energy management	Building/generic energy emissions [20–24, 26, 27, 30] Emissions specific equipement (Xray, operating room instruments,etc.) [24] Energy for cleaning [24] Energy use -length of stay [30] Energy use -bedsite [31] Energy use -remote equipment [31]	Yes^b^
	Transport	Freight transport [21, 22] Business travel [21, 22, 27]	Yes
	Procurement and products	Food and catering [21, 22] Business services [21, 22] Materials [20] Other procurement (non food) [21, 22]	Yes^b^
		Pharmaceuticals and chemicals [20–22] Paper products [22] Manufactured fuels, chemicals, gases [22] Other manufactured products [22]	Yes^b^
	Emissions from commissioned care	Health and social care services [21, 22]	No
Outcomes and Control	Outcomes	Water and sanitation [21, 22] Measurement and monitoring of performance^a^	Yes^b^
	Control	Accountability mechanisms such as reporting requirements [19] Desire for continuous improvement [19] Collaborative process to create internal policies and goals [19]	Yes^b^

^a^Identified as relevant in the parallel sub-project by medical doctors

^b^No assessment of these specific items but general assessment is included in the model

**Fig. 3:**
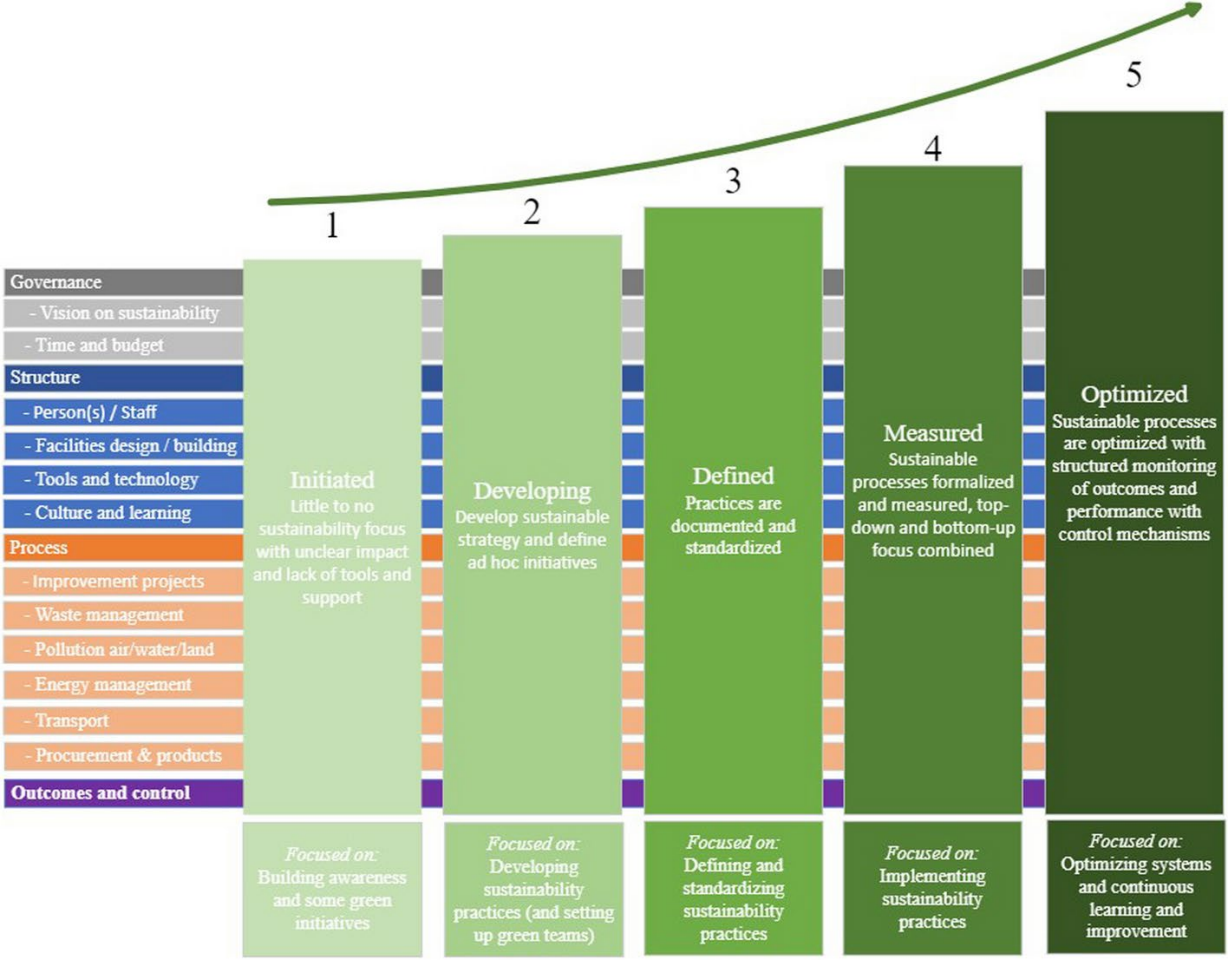
Overall design of maturity model concepts per domain and maturity levels at end of design phase

Table 2 demonstrates that several of the concepts mentioned in the literature were also deemed relevant to include in the model by medical doctors in the sub-project. Furthermore, these doctors identified six additional concepts. Although a number of concepts were omitted from the maturity model by the expert panel, the majority of concepts were agreed to be included, with some being suggested for inclusion at a more general level. Figure 3 summarizes the maturity model concepts selected by the expert panel in the domains: Governance, Structure, Process and Outcomes & Control. The hierarchy of the model with concepts and measurable items per domain is presented in Table 3.

**Table 3:** Maturity model hierarchy

**Domain**	**Concept**	**Measurable item** (all in relation to emvironmental sustainability)
**Governance**	G01.Vision on sustainability	G01.1 Management vision on sustainability
	G02. Time and budget	G02.1 Allocation of time
		G02.2 Allocation of budget
**Structure**	S01. Person(s) / Staff	S01.1 Visibility and recognition of green effort
		S01.2 Awareness on healthcare impact on environment
		S01.3 Knowledge on green practices
		S01.4 Occurrence of multidisciplinary teamwork
		S01.5 Intrinsic motivation to reduce environmental impact of daily work
		S01.6 Management of Change skills of people leading green initiatives
		S01.7 Communication between people leading green initiatives and others
	S02. Facilities design/building	S02.1 Building design and status (isolation, stairways, plants/trees, location close to public transport)
		S02.2 Installation of LED lights
		S02.3 Efficiency in use of Heating, Ventilation, Airconditioning, Cooling
		S02.4 Facilities for cleaning to reuse (sterilisation or washing)
	S03. Tasks	S03.1 Sustainability by default in standard tasks
		S03.2 Formalization of green actions in daily work
	S04. Tools and technology	S04.1 Availability of waste bins for seperate collection for glas, plastics, medical waste, general waste, paper and carbon
		S04.2 Availability of knowledge platform
		S04.3 Availability of sustainability scan
		S04.4 Availability and use of Green Dashboard
	S05. Organization of sustainability	S05.1 Sharing successes and lessons
		S05.2 Formal communication structure
		S05.3 Training and awareness plan
		S05.4 Availability of Green training
	S06. Culture	S06.1 Management commitment
		S06.2 Green communication
		S06.3 Green rules and procedures
		S06.4 Enabling environment
		S06.5 Personal involvement (participation)
**Process**	P01. Green improvement projects	P01.1 Running of improvement projects for sustainable healthcare
	P02. Waste management	P02.1 Waste management process with focus on environmental impact reduction
	P03. Pollution control - emissions to air	P03.1 Air pollution management process
	P04. Pollution control - emissions to land	P04.1 Land pollution management process
	P05. Pollution control - emissions to water	P05.1 Water pollution management process
	P06. Conservation of natural resources (eg. Water, food)	P06.1 Focus on reducing food waste
		P06.2 Focus on reducing water use / spillage
	P07. Energy management	P07.1 Energy management process with focus on environmental impact reduction
	P08. Transport	P08.1 Employee travel management process with focus on environmental impact reduction
		P08.2 Use of e-consultation to replace physical consultation
	P09. Procurement and products	P09.1 Use of recycled, reused or remanufactured products/materials
		P09.2 Reduce procurement and use of products (incl repair)
		P09.3 Inventory management to reduce out of date products
		P09.4 Level of circularity (Reuse, refurbish, recycle)
		P9.5 Efficient use to limit waste and reduce procurement volume
		P9.6 Procurement: Consideration of upstream environmental impact is considered when buying/ordering products or materials
**Outcomes & Control**	C01. Measuring of process performance	C01.1 Environmental impact measuring process
	C02. Monitoring of process performance	C02.1 Environmental impact monitoring process
	C03. Evaluation of process performance	C03.1 Environmental impact evaluation process
	C04. Adaptation of process performance	C04.1 Environmental impact improvement process

The average scores derived from evaluation surveys after testing the model in various healthcare settings are provided in Table 4. The different maturity model evaluation criteria were all graded with scores 3.3 to 4.7 on a 5-point Likert scale, indicating moderate to strong agreement with the maturity model’s sufficient level of Understandability, Ease of use, Usefulness and Practicality in general and within the maturity model’s categories.

**Table 4:** Scores of closed survey questions (scale 1 (strongly disagree) to 5 (strongly agree))

Subject:	Average score (standard deviation):
Governance	4.7 (0.06)
Outcomes & Control	4.4 (0.17)
Process	4.1 (0.13)
Structure	4.2 (0.10)
Ease of use	4.1 (0.13)
Maturity levels	3.3 (0.14)
Understandability	4.1 (0.08)
Usefulness and Practicality	3.9 (0.11)

No correlations between questionnaire subjects were found while the comparison of questionnaire scores between departments identified significant differences in the evaluation scores for the maturity model’s categories ‘Governance’ and ‘Outcomes and Control’. Table 5 presents a summary of comments and suggestions provided by respondents after testing the model.

**Table 5:** Suggestions for improvement resulting from testing the model in 6 case studies per domain

**Domain**	**Item**	**Comments/suggestions** N equals to the number of respondents providing the comment / suggestion
Governance	New measurement items	Add an assessment item on Green Team organization (*N* = 1) Add an assessment item on how departments carry out their sustainability processes (e.g. central / decentral) (*N* = 2)
Outcomes and Control	All	Make assessment items clearer by including examples (*N* = 2) Update the maturity level descriptions so that the steps between levels does not feel too big (*N* = 1)
Process	P03 Pollution control P09 Procurement and products	Specify descriptions of some assessment items to make them feel less similar or combine them (*N* = 4) Update maturity level descriptions so that the steps between levels does not feel too big (*N* = 1)
	New measurement item	Add assessment items for medication / medicinal waste (*N* = 3)
Structure	S05 Traning and awareness	Combine some assessment items (*N* = 1)
	S01.4 Occurance multidisciplinary teamwork S01.6 Management of Change skills S02.1 Building	Include examples for assessment items (*N* = 1)
All	Ease of Use	Change to one view to minimize the need to scroll (*N* = 1) Create a graph that automatically shows and updates the scores of the department (*N* = 1)
	Maturity Levels	Update some maturity level descriptions so that the steps between levels does not feel too big (*N* = 2) Update some maturity level descriptions to improve clarity (*N* = 1) *Keep in mind that if the department is performing half of the work of a maturity level description, the department gets the score of the lower maturity level (N* = *1)*
	Understandability	Include examples for assessment items (*N* = 2) Update some maturity level descriptions to improve clarity (*N* = 1)
	Usefulness and Practicality	*Consider the usefulness of assessment items that are similar to each other (N* = *3)* *Keep in mind that a department that is less sustainable than another department, but documents more, will score higher on the maturity model (N* = *4)*
	Other	*Keep in mind that adding more assessment items might make the model too specific (N* = *1)*

Based on these comments the maturity model was adjusted by the research team. The suggestion to add an assessment item on Green Team organization was not processed because Green Teams are considered one of many ways by which organizations can focus on environmental sustainability. There is no evidence that Green Team organization can be related to a higher or lower level of maturity. Changing to one view to minimize the need to scroll is not implemented because in the current software (Excel) this was not possible and the research team did not have resources for building the maturity model checklist in another software program. All other suggestions were incorporated in the resulting maturity model version 1.0 which is available in Appendix E.

## Discussion

The present study was designed to develop and test a maturity model for sustainable healthcare.

The results of this study show that the model developed is effective for identifying the current state of sustainability in terms of the environment in a standardized way. In all case studies the model was applied in less than one day through self-assessment by unexperienced practitioners who confirmed the model to be understandable, easy to use, useful and practical.

### Limitations and future research

Due to the relatively new nature of the subject of sustainable healthcare, the available literature provides limited information on the definition, measurement, and progression of maturity for sustainability in healthcare organizations. Therefore, it is a strength of this study that concepts identified from literature were combined with insights from medical doctors and an expert panel to develop the model.

Despite the promising results of this study, there is abundant room for enhancing the version 1.0 model it delivered by further investigating the model and its application in healthcare. We give five suggestions for future research related to the model.

The first suggestions result from comparing our maturity model to others. Even though our maturity model is the first maturity model focusing on sustainable healthcare, it is not the first maturity model developed for healthcare. For instance, a study by Gomes and Romão [32] found 26 maturity models specifically for health information systems. All these models define increasing capability for a specific focus, such as the maturity of process-orientation or infrastructure. One of the most recent maturity models developed for the healthcare setting, is the maturity model for Internet of Things (IoT) adoption in hospitals from Hasić et al. [33]. Like our model, the maturity model for IoT contains aspects for the categories ‘Governance’ and ‘Monitoring’. A difference of the IoT model compared to our model is that it is more specific in its use of categories. For example, while our model uses the rather generic domains ‘Structure’ and ‘Process’ to cluster concepts, the IoT model contains more specific categories such as a category focusing solely on one specific process (e.g. ‘Data Governance’) or structure (e.g. ‘Organisational culture’). Even though participants in the current study did not comment on the categorization of concepts, future research could investigate if the use of more specific domains improves the efficacy of the model. For instance, by defining domains for key processes such as those defined by WHO in their guide for environmentally sustainable healthcare facilities such as communications and awareness or monitoring of energy, water and waste [34].

Another difference between our maturity model and other healthcare maturity models, like the widely accepted Capability Maturity Model (CMMI) for software development [35] is in the main focus of the models. Models such as CMMI place predominant emphasis on processes and the inputs and outputs associated with them, whereas our model encompasses organizational structures, processes, and outputs for a comprehensive perspective. Our model does not define inputs, and it does not link outcomes to particular activities. The focus on inputs and outputs was also used by Cimprich et al. [20] in their paper included in the literature review in our research. Therefore, we suggest to perform further research to better understand if adding inputs and connecting processes with inputs and outputs could improve the model.

According to Wendler [15], the user of a maturity model has to decide which maturity level is most appropriate to aim for in a given situation. This decision making on setting maturity goals was not investigated in the current study. Even though it could be tempting to state that healthcare organizations should always aim for the highest level of maturity to achieve the lowest possible impact on the environment, other aspects such as patient health outcomes and financial implications also need to be considered when reforming healthcare operations [36]. Hence, a third topic for future research is on defining criteria that can be used to select the best maturity level to aim for in a specific situation.

Though the initial maturity model was tested and enhanced in case studies, the validation of the models’ instrument reliability and generalizability in more detail is a fourth topic for future research. This validation could be done by performing an inter-rater reliability test and by applying the model in other healthcare settings such as primary care or other countries than the Netherlands.

This 1.0 version of a maturity model for sustainable healthcare focuses on assessing the environmental impact of healthcare without addressing resilience to climate change. In their guidance for climate-resilient and environmentally sustainable healthcare facilities, WHO call for healthcare organizations to also improve their resilience besides reducing their environmental impact [34]. Future research could focus on repeating our study while focusing on healthcare organization’s maturity in terms of resilience to climate change and aim to develop a separate maturity model to address resilience or to integrate it in the current model.

Apart from the further development and validation of the maturity model, the approach used to identify improvement actions in this study should also be further tested and refined to determine the most effective method for using the model to drive improvements. While the approach followed in this research—consisting of a root cause analysis where researchers and staff collaboratively identified underlying issues for lower scores, followed by brainstorming and assessing the feasibility of potential solutions—appeared to work well, it may not necessarily be the most optimal method in all cases. It is important to investigate whether this approach is universally applicable or if alternative approaches might be more effective, especially across different organizational settings. Future research should focus on exploring various methods to establish the most efficient approach for identifying and implementing improvement actions tailored to diverse organizational contexts.

### Implications

As suggested by De Bruin et al. [16], by publishing the model with this paper we make it widely available to allow for reviewing the model’s generalizability and to collect feedback to continue improving the model. The model can be used by healthcare professionals with different roles, such as quality improvement professionals, managers, nurses or clinicians who have ambitions for improving the environmental sustainability at their workplace or broader organisation. As experienced in our six case studies, implementing the model does not require specific training or knowledge. If preferred, background information on healthcare’s impact on the environment can be retrieved from sources like the guidance from WHO [34]. Based on experiences and recommendations on environmental sustainability improvement in healthcare [9, 34] we recommended to involve different disciplines when implementing the model in healthcare organizations. Diverse disciplines working together when using the maturity model can improve the validity of model results, reduce implementation resistance, and raise awareness of the environmental impact throughout the healthcare organization. Besides healthcare professionals involved in patient care, procurement and waste management professionals should be consulted when implementing the model.

The model has the potential to be integrated into routine quality monitoring programs, which would help ongoing and long-term improvement initiatives, in addition to being used as a one-time tool to obtain insights into the present status and identify improvement activities. This is consistent with the perspective of Mortimer et al. [36], who view environmental impact as a critical component of healthcare quality.

Healthcare communities could adopt the model and include it in their best practice repository or reference the model in their guidelines and policies as a tool to support environmental sustainability improvement initiatives. This can increase the awareness of healthcare professionals on the environmental impact of healthcare and motivate healthcare professionals to adopt and integrate it into their own systems.

## Conclusions

This research aimed to develop a maturity model for assessing the current state and possible next steps with regard to environmental sustainability in healthcare settings.

To design, populate and test the model, concepts were extracted from literature and inputs from medical doctors were combined with an expert panel and six case studies in Dutch healthcare facilities.

Results indicate that the maturity model developed in this study could be easily applied by healthcare practitioners without training in a short timeframe of several hours and result in valuable insights in the current status with regard to environment footprint.

As there is an urgent need to mitigate climate impact and reduce the substantial environmental impact of healthcare, the model developed in this study can be implemented systematically to support healthcare in becoming more sustainable. Experiences from these implementations can be a valuable source for continued evaluation and improvement of the maturity model.

## Supplementary Information

Below is the link to the electronic supplementary material.

Supplementary Material 1.

## Data Availability

The datasets used and analysed during the current study are available from the corresponding author on reasonable request.
