# Supplementary material for: Linking theory and practice to advance sustainable healthcare: the development of maturity model version 1.0
Source: BMC Health Serv Res. 2024 Nov 5;24:1350. doi: 10.1186/s12913-024-11749-8 (PMC11539419; doi:10.1186/s12913-024-11749-8)
Supplement: Supplementary file 1 — Supplementary Material 1. [file 12913_2024_11749_MOESM1_ESM.zip › Appendix.docx]

Appendix A. Maturity model design decisions

Table A1: Design decisions

| **Criterion** | **Design decision on characteristic** |
| --- | --- |
| Audience | Internal, management and healthcare practitioners (e.g., members of Green Teams) |
| Method of application | Self-assessment |
| Driver of application | Internal requirement |
| Respondents | Staff (healthcare professionals) |
| Application | Multiple entities ((subsets of) healthcare organizations) / single region (The Netherlands) |
| Approach for defining stages | Top-down |
| Reporting of stages | Stage-gate approach with separate assessments for domain components and sub-components |

Appendix B. RAPID review protocol summary

**Objective:** Identify key constructs most common to describe the environmental impact of healthcare

**PICO:**

- PICO question: What are the organizational, people and process factors that influence the application of pressures by healthcare organizations on the ecological environment in terms of resource usage, pollution, and generation of wast?
- Population – all healthcare facilities including hospitals, pharmacies, mental healthcare institutions, elderly care etc.
- Intervention – factors that influence the application of pressures on the environment in terms of resource usage, generation of waste and pollution of air, water and soil.
- Comparator: Not applicable
- Outcomes: Identification of concepts that influence the application of pressures on the environment in terms of resource usage, pollution, and generation of waste in healthcare facilities.

**Seach query:** (footprint) AND ((healthcare) OR (health care)) AND (framework OR model) in Title/Abstract

EBSCO query: AB ((footprint) AND ((healthcare) OR (health care)) AND (framework OR model))

**Inclusion criteria:** Peer Reviewed, English language, all dates up until date of search

**Exclusion criteria**: No primary research, not describing environmental impact of healthcare, no model or framework presented, article not available

**Date:** The search was conducted on March 1st 2023.

**Databases:** Cochrane CENTRAL, MEDLINE, Academic Search Premier (EBSCO)

Appendix C. Maturity model evaluation survey

| **Criteria** | **Score (1-5)** *1 = Strongly disagree 2 = Slightly disagree 3 = Neither disagree nor agree 4 = Slightly agree 5 = Strongly agree* |
| --- | --- |
| *Understandability* |  |
| **The maturity levels are understandable** |  |
| T**he assessment items are understandable** |  |
| **The documentation is understandable** |  |
| *Ease of use* |  |
| **The scoring scheme is easy to use** |  |
| **The assessment items are easy to use** |  |
| **The documentation is easy to use** |  |
| *Usefulness and practicality* |  |
| **The maturity model is useful for conducting assessments** |  |
| **The maturity model gives a complete representation of the sustainability of the assessed department** |  |
| **The maturity model is practical for use in practice** |  |
| *Maturity levels* |  |
| **The maturity levels are sufficient to represent all maturation stages of the domain** |  |
| **There is no overlap detected between descriptions of maturity levels** |  |
| **Assessment domains** *(Score each seperate domain of the model)* |  |
| *Checklist Governance* |  |
| **The assessment items are relevant to this domain** |  |
| **The assessment items cover all aspects impacting/ involved in the domain** |  |
| **The assessment items are clearly distinct** |  |
| **The assessment items are correctly assigned to their respective maturity level** |  |
| *Checklist Structure* |  |
| **The assessment items are relevant to this domain** |  |
| **The assessment items cover all aspects impacting/ involved in the domain** |  |
| **The assessment items are clearly distinct** |  |
| **The assessment items are correctly assigned to their respective maturity level** |  |
| *Checklist Process* |  |
| **The assessment items are relevant to this domain** |  |
| **The assessment items cover all aspects impacting/ involved in the domain** |  |
| **The assessment items are clearly distinct** |  |
| **The assessment items are correctly assigned to their respective maturity level** |  |
| *Checklist Outcomes & Control* |  |
| **The assessment items are relevant to this domain** |  |
| **The assessment items cover all aspects impacting/ involved in the domain** |  |
| **The assessment items are clearly distinct** |  |
| **The assessment items are correctly assigned to their respective maturity level** |  |
|  |  |
| **Open questions** |  |
| **Would you update the maturity level description? If so please explain what and why?** |  |
| **Would you update any of the assessment items? If so please explain what and why?** |  |
| **Would you add any assessment items? If so please explain what and why?** |  |
| **Would you remove any of the assessment items? If so please explain what and why?** |  |
| **Do you have any other suggestions for improvement?** |  |

Appendix D Literature overview capabilities and measurement criteria

| Source | Study setting, location | Concept | Sub-concept | Comments |
| --- | --- | --- | --- | --- |
| Tennison et al., 2021[^21^] | National healthcare setting, United Kingdom | Energy management | On-site fuel use  Electricity use  Anaesthetic gases  Water  Waste  Metered dose inhalers | Focus on Greenhouse Gas emissions |
| Tennison et al., 2021[^21^] | National healthcare setting, UK | Person(s) / Staff | Staff commute  Patient travel  Visitor travel | Focus on Greenhouse Gas emissions |
| Tennison et al., 2021[^21^] | National healthcare setting, United Kingdom | Procurement and products | Pharmaceuticals and chemicals  Medical equipment  Non-medical equipment  Business services  Food and catering  Other procurement | Focus on Greenhouse Gas emissions |
| Tennison et al., 2021[^21^] | National healthcare setting, United Kingdom | Transport | Business Travel | Focus on Greenhouse Gas emissions |
| Tennison et al., 2021[^21^] | National healthcare setting, United Kingdom | Emissions from commissioned care | Commissioned healthcare services | Focus on Greenhouse Gas emissions |
| Monsell et al., 2021[^22^] | Mental health care delivery, general | Pollution air/water/land | Anaesthetic gases |  |
| Monsell et al., 2021[^22^] | Mental health care delivery, general | Facilities design/building | Construction |  |
| Monsell et al., 2021[^22^] | Mental health care delivery, general | Tools and technology | ICT  Medical equipment  Non-medical equipment |  |
| Monsell et al., 2021[^22^] | Mental health care delivery, general | Waste management | Waste products and recycling |  |
| Monsell et al., 2021[^22^] | Mental health care delivery, general | Outcomes | Water and sanitation |  |
| Monsell et al., 2021[^22^] | Mental health care delivery, general | Procurement and products | Pharmaceuticals and chemicals  Business services  Food and catering  Other procurement (non food) |  |
| Monsell et al., 2021[^22^] | Mental health care delivery, global | Emissions from commissioned healthcare | Commissioned healthcare services |  |
| Monsell et al., 2021[^22^] | Mental health care delivery, global | Building | Construction |  |
| Woolen et al., 2023[^27^] | Radiology, USA | Waste management | Solid Waste  Diagnostic imaging waste |  |
| Woolen et al., 2023[^27^] | Radiology, USA | Energy Management | Electricity use |  |
| Woolen et al., 2023[^27^] | Radiology, USA | Transport | Business travel (airplane) |  |
| Soares et al., 2023[^23^] | All healthcare, general | Waste management | Waste products and recycling | Focus on circular economy and greenhouse gas emissions |
| Soares et al., 2023[^23^] | All healthcare, general | Energy Management | Energy usage | Focus on greenhouse gas emissions |
| Soares et al., 2023[^23^] | All healthcare, general | Tools and technology | Telemedicine | Focus on greenhouse gas emissions |
| Soares et al., 2023[^23^] | All healthcare, general | Person(s)/staff | Behaviour or engagement  Staff commute  Patient travel  Visitor travel |  |
| Soares et al., 2023[^23^] | All healthcare, general | Improvement projects | Sustainability improvement projects |  |
| Pollard et al., 2013[^30^] | National health services, UK | Energy management | Energy use  Length of stay |  |
| Pollard et al., 2013[^30^] | National health services, UK | Person(s)/staff | Patient travel |  |
| Pollard et al., 2014[^31^] | Critical Care Unit, UK | Energy management | Energy use – bedsite  Energy use – remote equipment | Bedsite energy use to power devices for organ support and patient monitoring |
| Cimprich et al., 2019[^20^] | Healthcare, general | External - Laws and regulations | Laws and regulations |  |
| Cimprich et al., 2019[^20^] | Healthcare, global | Energy management | Energy |  |
| Cimprich et al., 2019[^20^] | Healthcare, global | Procurement and products | Materials  Chemicals |  |
| Cimprich et al., 2019[^20^] | Healthcare, global | Tools and technology | Devices  Equipment |  |
| Cimprich et al., 2019[^20^] | Healthcare, global | Facilities design/building | Infrastructure |  |
| Cimprich et al., 2019[^20^] | Healthcare, general | Waste management | Waste streams  Reprocessing of used medical devices |  |
| Power et al., 2012[^28^] | Minimally invasive surgery, US | Pollution air/water/land | Anaesthetic gases | Waste specified in disposable laparoscopic trocars, plastic and instruments |
| Power et al., 2012[^28^] | Minimally invasive surgery, US | Waste management | Waste products and recycling | Waste specified in disposable laparoscopic trocars, plastic and instruments |
| Carino et al., 2022[^19^] | Hospital food services, general | Culture | Beliefs  Feelings of importance  Feelings of responsibility  Commitment for driving change  Staff feels comfortable initiating change  Shared desire to inspire other employees and institutions  Ambition to receive awards and recognition |  |
| Carino et al., 2022[^19^] | Hospital food services, general | Governance – Time and budget | Investment in roles dedicated to sustainability |  |
| Carino et al., 2022[^19^] | Hospital food services, general | Governance – Vision on sustainability | Focus on long term impacts in decision making  Sense of obligation to be a leader within the community  Environmental sustainability integrated in identity and mission |  |
| Carino et al., 2022[^19^] | Hospital food services, general | Culture | Executive Support of staff ideas |  |
| Carino et al., 2022[^19^] | Hospital food services, general | Control | - Accountability mechanisms such as reporting requirements - Desire for continuous improvement - Collaborative process to create internal policies and goals |  |
| Carino et al., 2022[^19^] | Hospital food services, general | Person(s)/Staff | Teams or departments dedicated to sustainability |  |
| Carino et al., 2022[^19^] | Hospital food services, general | Laws and regulations | Laws and regulations | E.g. for procurement, restrictive |
| Carino et al., 2022[^19^] | Hospital food services, general | Partnerships | Work with other hospitals to influence government policy |  |
| Duane et al., 2014[^24^] | National healthcare setting, United Kingdom | Energy management | Emissions from:  Xray  Equipment general  Light  Instruments operating room  Cleaning  Generic energy consumption |  |
| Duane et al., 2014[^24^] | National healthcare setting, United Kingdom | Person(s)/Staff | Patient travel |  |
| Andrews et al., 2013[^25^] | National healthcare setting, UK | Person(s)/Staff | Patient travel | Travel differentiated by type (car, bus, taxi, walk, other) |
| Narayanan et al., 2022[^29^] | Pediatric anaesthesia, UK | Pollution air/water/land | Anaesthetic gases |  |
| Lokmic-Tomkins et al, 2022[^26^] | Digital health interventions, general | Tools and technology | ICT  Telemedicine |  |
| Lokmic-Tomkins et al, 2022[^26^] | Digital health interventions, general | Energy management | Power consumption |  |
| Lokmic-Tomkins et al, 2022[^26^] | Digital health interventions, general | Person(s)/Staff | Patient travel |  |

Appendix E Maturity Model version 1.0

Excel file with the maturity model
